# Metagenome-assembled genome of *Oscillospiraceae* bacterium strain ZGZL, an anaerobic chloromethane-degrading bacterium enriched from rice paddy soil

**DOI:** 10.1128/mra.00287-26

**Published:** 2026-06-22

**Authors:** Xin Wang, Hongyan Wang, Xuhao Wang, Manman Zhang, Yiru Cui, Hengyi Liao, Jiaojiao Yang, Yangyang Zou, Lisi Jiang, Xiuying Li, Yi Yang

**Affiliations:** 1 Key Laboratory of Pollution Ecology and Environmental Engineering, Institute of Applied Ecology, Chinese Academy of Sciences 74763, Shenyang, Liaoning, China; 2 University of Chinese Academy of Sciences 74519, Beijing, China; 3 Water Resources Research Center, University of Hawaii at Manoa, Honolulu, Hawaii, USA; 4 Department of Civil, Environmental and Construction Engineering, University of Hawaii at Manoa, Honolulu, Hawaii, USA; 5 School of Pharmaceutical Engineering, Shenyang Pharmaceutical University 58575, Shenyang, Liaoning, China; 6 College of Life Sciences, Shenyang Normal University 12402, Shenyang, Liaoning, China; 7 Key Laboratory of Forest Ecology and Silviculture, Institute of Applied Ecology, Chinese Academy of Sciences 74763, Shenyang, Liaoning, China; University of Wisconsin-Madison, Madison, Wisconsin, USA

**Keywords:** *Oscillospiraceae*, chloromethane, rice paddy soil, anaerobes, biodegradation

## Abstract

*Oscillospiraceae* sp. strain ZGZL is an anaerobic bacterium capable of degrading chloromethane. Here, we report the metagenome-assembled genome sequence of strain ZGZL, which has a genome size of 2.04 Mb and a G+C content of 52.56%.

## ANNOUNCEMENT

Metagenome-assembled genome (MAG) ZGZL was identified from an anaerobic chloromethane (CM)-degrading enrichment culture established from rice paddy soil. Surface soil samples (0–10 cm depth) were collected from a rice paddy field in Jianli City, Hubei Province, China (29°58′N, 112°53′E) using sterile spatulas. The samples were immediately transferred to sterile polypropylene tubes, transported to the laboratory on ice, and stored at 4°C prior to enrichment cultivation. Cultivation was performed in 120-mL glass serum bottles containing 80 mL of a bicarbonate-buffered (30 mM) mineral salts medium under an N₂/CO₂ headspace, prepared according to established protocols (1–3). The ZGZL-dominated enrichment culture anaerobically converted 50 µmol of CM primarily to acetate, with minor amounts of formate detected. Metagenomic sequencing was performed on the enrichment culture, and the genome of strain ZGZL was recovered as a MAG through metagenomic assembly and binning. This genomic resource is highly relevant, as the anaerobic biodegradation of CM remains poorly understood, with *Acetobacterium dehalogenans* strain MC being one of the few strictly anaerobic CM-degrading isolates reported since 1991 (4).

Genomic DNA was extracted using the E.Z.N.A. Stool DNA Kit (Omega Bio-Tek, Norcross, GA, USA) according to the manufacturer’s instructions. Illumina sequencing libraries were prepared using the Nextera XT DNA Library Preparation Kit and sequenced on an Illumina NovaSeq 6000 platform (Illumina, San Diego, CA, USA) with 150-bp paired-end reads (5). A total of 76,895,262 high-quality paired-end reads (2 × 150 bp) were obtained after quality filtering with Trimmomatic v0.36 and assembled using metaSPAdes v3.15.3 (6, 7). Because sequencing was performed on a mixed anaerobic enrichment culture rather than a pure isolate, metagenomic binning was required to recover the genome of strain ZGZL from the community assembly. Genome binning was performed using MaxBin2 v2.2.4, and taxonomic classification was carried out with GTDB-Tk v2.3.2 (8, 9). Genome completeness and contamination were assessed using CheckM v1.1.3 (10) with default parameters. Functional annotation was performed using the NCBI Prokaryotic Genome Annotation Pipeline (PGAP) (11).

The near-complete draft genome of strain ZGZL consists of a single MAG of 2,036,933 bp with a G+C content of 52.56%. Genome annotation identified 1,978 genes and partial rRNA operons, including three 5S rRNA genes, two 16S rRNA genes, one 23S rRNA gene, 1,916 protein-coding sequences, and 48 tRNA genes. Annotation analyses revealed an incomplete Wood–Ljungdahl pathway, in which three methyltransferases and multiple corrinoid-dependent methyl group transfer proteins were encoded, whereas key enzymes involved in the formate-generating step of the methyl branch were absent. While this genomic configuration implies that strain ZGZL relies on corrinoid-mediated methyl transfer reactions rather than a canonical Wood–Ljungdahl pathway for C1 metabolism, further investigation is required to confirm whether this reflects its true biological capacity or is an artifact of incomplete MAG recovery. Furthermore, comparative genomic analysis showed that strain ZGZL is only distantly related to *Sporobacter termitidis* DSM 10068 (GenBank accession number FQXV00000000). The average nucleotide identity (ANI) and digital DNA–DNA hybridization (dDDH) values were 72.54% and 13.90%, respectively, indicating that strain ZGZL may represent a novel genus-level lineage. Collectively, these genomic features provide insights into the potential metabolic basis of CM utilization by strain ZGZL in anoxic environments.

## Data Availability

The metagenome-assembled genome of *Oscillospiraceae* sp. strain ZGZL has been deposited in GenBank under the accession number GCA_038405045.1. The raw sequencing reads have been deposited in the Sequence Read Archive (SRA) under accession number SRR34078185. The associated BioSample and BioProject accession numbers are SAMN40562231 and PRJNA1090301, respectively.

## References

[1] Wang X, Wang H, Jin H, Liao H, Qian X, Zhang M, Weng Z, Hoffnagle E, Wang X, Yang J, Wang J, Cui Y, Li X, Liu X, Chen X, Yang Y. 2025. Complete genome sequence of Rhodococcus qingshengii strain R isolated from Antarctic soil. Microbiol Resour Announc 14:e0131624. doi:10.1128/mra.01316-24 40326773 PMC12160466

[2] Löffler FE, Sanford RA, Ritalahti KM. 2005. Enrichment, cultivation, and detection of reductively dechlorinating bacteria. Meth Enzymol 397:77–111. doi:10.1016/S0076-6879(05)97005-5 16260286

[3] Wang H, Wang X, Huang S, Yang S, Liao H, Wang X, Jin H, Wang J, Li X, Yan J, Yang Y. 2025. Complete genome sequence of “ Candidatus Dehalogenimonas loeffleri” strain W, a highly salt-tolerant chlorinated alkane-dechlorinating bacterium isolated from estuarine sediments. Microbiol Resour Announc 14:e0031924. doi:10.1128/mra.00319-24 39601548 PMC11737167

[4] Traunecker J, Preuß A, Diekert G. 1991. Isolation and characterization of a methyl chloride utilizing, strictly anaerobic bacterium. Arch Microbiol 156:416–421. doi:10.1007/BF00248720

[5] Cui Y, Yang Y, Li X, Wang J, Jin H, Liao H, Wang X, Shi K, Huang S, Zhou T, Löffler FE, Yan J. 2025. Complete genome sequence of Dehalococcoides mccartyi strain NK, an acid-tolerant organohalide-respiring bacterium. Microbiol Resour Announc 14:e0063325. doi:10.1128/mra.00633-25 40788149 PMC12424303

[6] Bolger AM, Lohse M, Usadel B. 2014. Trimmomatic: a flexible trimmer for Illumina sequence data. Bioinformatics 30:2114–2120. doi:10.1093/bioinformatics/btu170 24695404 PMC4103590

[7] Nurk S, Meleshko D, Korobeynikov A, Pevzner PA. 2017. metaSPAdes: a new versatile metagenomic assembler. Genome Res 27:824–834. doi:10.1101/gr.213959.116 28298430 PMC5411777

[8] Wu Y-W, Simmons BA, Singer SW. 2016. MaxBin 2.0: an automated binning algorithm to recover genomes from multiple metagenomic datasets. Bioinformatics 32:605–607. doi:10.1093/bioinformatics/btv638 26515820

[9] Chaumeil P-A, Mussig AJ, Hugenholtz P, Parks DH. 2022. GTDB-Tk v2: memory friendly classification with the genome taxonomy database. Bioinformatics 38:5315–5316. doi:10.1093/bioinformatics/btac672 36218463 PMC9710552

[10] Parks DH, Imelfort M, Skennerton CT, Hugenholtz P, Tyson GW. 2015. CheckM: assessing the quality of microbial genomes recovered from isolates, single cells, and metagenomes. Genome Res 25:1043–1055. doi:10.1101/gr.186072.114 25977477 PMC4484387

[11] Tatusova T, DiCuccio M, Badretdin A, Chetvernin V, Nawrocki EP, Zaslavsky L, Lomsadze A, Pruitt KD, Borodovsky M, Ostell J. 2016. NCBI prokaryotic genome annotation pipeline. Nucleic Acids Res 44:6614–6624. doi:10.1093/nar/gkw569 27342282 PMC5001611

